# Fibroblast Dynamics in Keloid Pathogenesis: Unraveling Cellular Crosstalk and Novel Therapeutic Targets

**DOI:** 10.1155/drp/2528205

**Published:** 2026-01-27

**Authors:** Ziad Alkouz, Ala’a Al Suwait, Lian Zhang, Rehab Alhejairi, Freddy Gahimbare, Mahmoud Qalalwa, Bin Yang

**Affiliations:** ^1^ Dermatology Hospital of Southern Medical University, Guangzhou, Guangdong, China, fimmu.com

**Keywords:** cellular heterogeneity, fibroblast dynamics, keloid fibroblasts, precision medicine, TGF-β signaling, therapeutic targets

## Abstract

Keloid scars represent a complex fibroproliferative disorder characterized by abnormal wound healing and excessive collagen deposition. Central to keloid pathogenesis are dynamic fibroblast populations that undergo extensive phenotypic transitions, including heterogeneous subpopulation differentiation, enhanced migration, myofibroblast transdifferentiation, and sustained activation states. This review examines fibroblast dynamics as the central orchestrator of keloid formation, analyzing how these cells interact with keratinocytes, immune cells, endothelial cells, and melanocytes to drive pathological scarring. We focus on key signaling pathways that directly regulate fibroblast function, including TGF‐β/Smad, VEGF, Wnt, and emerging regulators such as miR‐3606‐3p that integrate multiple fibrotic cascades. Current therapeutic approaches show variable efficacy, with surgical excision alone resulting in 45%–100% recurrence rates, while combination therapies incorporating radiation, intralesional injections, and novel molecular targets achieve improved outcomes. Emerging strategies include COX‐2 inhibition for dual antiproliferative and proapoptotic effects on keloid fibroblasts, stem cell therapies, and precision medicine approaches based on molecular profiling. Through deeper understanding of fibroblast dynamics and their regulatory networks, more effective therapeutic strategies can be developed to improve patient outcomes and quality of life.

## 1. Introduction

Keloid scars are a challenging condition in dermatology and plastic surgery. These scars develop when the body produces an excessive healing response after skin injury [1]. Epidemiological studies show keloids affect between 4% and 16% of all people. Evidence has confirmed a higher prevalence in people with darker skin tones, with rates up to 16% in African populations [2–5]. This racial predisposition has been well documented through multiple studies examining genetic and environmental factors [6, 7]. Furthermore, keloids show a strong familial tendency, with heritability rates of 72% for first‐degree relatives in certain populations [8].

Keloids significantly impact patient quality of life. In a comprehensive study of 100 keloid patients, 87% reported pain and itching, while 91% experienced movement restriction [9]. Psychological assessments reveal that 75% of keloid patients suffer from anxiety or depression [10, 11]. A recent meta‐analysis found that 82% of patients report difficulties in social interactions [12].

Recent molecular studies have revealed that keloid fibroblasts serve as the central orchestrators of pathological scar formation. Single‐cell RNA sequencing has identified distinct populations of keloid fibroblasts with specialized functions [13–15]. These keloid fibroblasts exhibit altered behavior compared to normal dermal fibroblasts, including enhanced proliferation, increased collagen synthesis, and resistance to apoptosis [16]. Understanding the dynamic nature of keloid fibroblasts and their interactions with other cell types is crucial for developing effective treatments.

## 2. Terminology and Definitions

To ensure clarity and consistency throughout this review, we define key terms.

Keloid fibroblasts: Fibroblasts derived from keloid tissue, exhibiting distinct phenotypic characteristics including enhanced proliferation, increased collagen synthesis, and resistance to apoptosis. This term encompasses all fibroblast subpopulations within keloid tissue.

Normal dermal fibroblasts: Fibroblasts from unaffected skin serving as controls in comparative studies.

Myofibroblasts: Activated keloid fibroblasts expressing α‐smooth muscle actin (α‐SMA) and exhibiting contractile properties.

Fibroblast subpopulations identified through single‐cell RNA sequencing:

• Inflammatory fibroblasts (expressing IL‐6, CCL2, and CXCL12).
• Matrix‐producing fibroblasts (expressing COL1A1, COL3A1, and fibronectin).
• Contractile myofibroblasts (expressing ACTA2 and smooth muscle myosin).
• Proliferative fibroblasts (expressing Ki67 and cyclin D1).

## 3. Fibroblast Dynamics: The Central Orchestrators of Keloid Formation

### 3.1. Fibroblast Subpopulation Heterogeneity

Keloid fibroblasts represent a heterogeneous population exhibiting distinct dynamic behaviors that drive pathological scar formation. Single‐cell RNA sequencing studies have revolutionized our understanding, revealing at least four distinct keloid fibroblast subpopulations [17, 18].

Inflammatory fibroblasts, expressing high levels of IL‐6, CCL2, and CXCL12, predominate at the keloid periphery where they maintain chronic inflammation. Matrix‐producing fibroblasts, characterized by elevated COL1A1, COL3A1, and fibronectin expression, concentrate in central regions, continuously depositing excessive collagen. Contractile myofibroblasts positive for α‐SMA align along tension lines, contributing to keloid contraction. Proliferative fibroblasts showing increased Ki67 and cyclin D1 expression distribute throughout the tissue [19, 20].

### 3.2. Migration and Invasion Dynamics

Keloid fibroblasts exhibit enhanced migratory capacity compared to normal dermal fibroblasts. This increased motility involves upregulation of matrix metalloproteinases (MMPs), particularly MMP‐2 and MMP‐9, enhanced integrin expression, especially α1β1 and α2β1 integrins, and altered cytoskeletal organization with increased stress fiber formation [21, 22].

The migratory behavior of keloid fibroblasts follows specific temporal patterns. In the early inflammatory phase (0–3 days postinjury), keloid fibroblasts migrate toward the wound site at 50% greater velocity than normal dermal fibroblasts. During the proliferative phase (3–21 days), they exhibit radial migration from the central keloid toward the periphery. In chronic keloids (> 6 months), migration becomes organized along collagen fiber orientations [23].

### 3.3. Transdifferentiation Processes

The conversion of normal fibroblasts to myofibroblasts represents a critical dynamic process in keloid formation. This transdifferentiation in keloid fibroblasts involves initial activation by TGF‐β1 and mechanical stress, acquisition of stress fibers, development of specialized cell–matrix adhesions, and increased contractile capacity [24, 25].

Keloid fibroblasts show resistance to apoptosis signals that normally terminate wound healing. This resistance involves upregulation of antiapoptotic proteins like Bcl‐2 and survivin, with downregulation of proapoptotic factors such as Bax and p53 [26, 27].

### 3.4. Activation State Transitions

Keloid fibroblasts undergo dynamic changes in activation states throughout disease progression. Quiescent fibroblasts in normal skin maintain tissue homeostasis. Upon injury, these cells transition to activated fibroblasts that increase proliferation and matrix synthesis. In keloids, fibroblasts become hyperactivated with loss of normal regulatory feedback mechanisms [28, 29].

The transition between activation states in keloid fibroblasts involves epigenetic modifications including DNA methylation changes and histone modifications that create stable but potentially reversible cellular phenotypes [30, 31].

### 3.5. Metabolic Dynamics

Keloid fibroblasts exhibit significant metabolic reprogramming. They demonstrate enhanced glycolysis similar to cancer cells, increased amino acid metabolism supporting collagen synthesis, and altered lipid metabolism affecting membrane composition and signaling [32]. These metabolic changes in keloid fibroblasts support the high energy demands of excessive matrix production.

## 4. Fibroblast‐Mediated Cellular Crosstalk in Keloid Formation

### 4.1. Fibroblast–Keratinocyte Interactions

Keloid fibroblasts maintain bidirectional communication with keratinocytes that amplifies pathological scarring. Keratinocytes in keloid tissue release twice the amount of IL‐6 and TNF‐α compared to normal keratinocytes, directly stimulating keloid fibroblast proliferation and collagen production [33]. In response, keloid fibroblasts secrete factors that promote keratinocyte proliferation and epithelial–mesenchymal transition [34].

Coculture studies demonstrate that keloid keratinocytes induce normal fibroblasts to adopt keloid‐like characteristics, while keloid fibroblasts can alter normal keratinocyte behavior [35]. This creates a self‐reinforcing loop where both cell types perpetuate the pathological state.

Figure 1 illustrates the complex network of cellular interactions centered on keloid fibroblasts, showing how multiple cell types contribute to the pathological microenvironment through paracrine signaling and direct cell–cell contact.

**Figure FIGURE 1:**
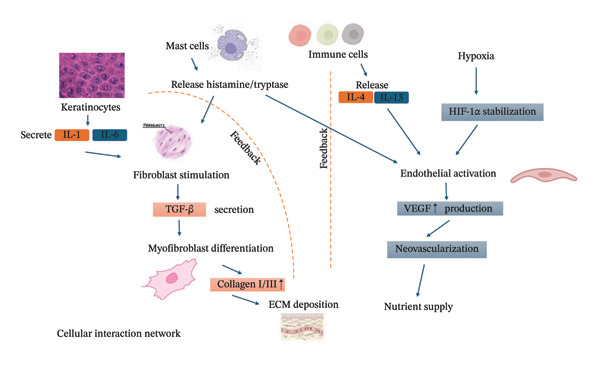
Cellular interaction network diagram.

### 4.2. Fibroblast–Immune Cell Interactions

Keloid fibroblasts actively recruit and modulate immune cells through chemokine secretion. They produce elevated levels of CCL2, recruiting monocytes that differentiate into profibrotic M2 macrophages [36]. These macrophages, in turn, release TGF‐β and IL‐13, further activating keloid fibroblasts [37].

T cells, particularly CD4+ cells, directly interact with keloid fibroblasts through cell–cell contact and cytokine signaling. Keloid fibroblasts express increased levels of ICAM‐1 and VCAM‐1, facilitating T cell adhesion [38]. The resulting IL‐4 and IL‐13 production by T cells enhances keloid fibroblast collagen synthesis by 40% [39].

Mast cells appear in higher numbers around keloid fibroblasts, releasing histamine and tryptase that stimulate fibroblast proliferation [40]. The number of mast cells correlates with keloid fibroblast density and collagen deposition [41].

### 4.3. Fibroblast–Endothelial Cell Interactions

Keloid fibroblasts produce 50% more VEGF than normal fibroblasts, promoting angiogenesis [42]. This increased vascularization supports keloid fibroblast survival and proliferation by ensuring adequate nutrient supply [43]. Endothelial cells undergo endothelial‐to‐mesenchymal transition in response to keloid fibroblast–derived TGF‐β, contributing to the expanded fibroblast pool [44].

### 4.4. Fibroblast–Melanocyte Interactions

Emerging evidence reveals that melanocytes influence keloid fibroblast behavior through iron‐mediated mechanisms. Increased melanin production induces iron overload and confers ferroptosis resistance to keloid fibroblasts [45]. This disrupts normal cell death pathways and enables keloid fibroblast survival despite high oxidative stress [46]. The iron–melanin complex may represent both a biomarker and therapeutic target for modulating keloid fibroblast function [47]. The complex network of cellular interactions in keloid pathogenesis is summarized in Figure 2. This comprehensive model illustrates how keloid fibroblasts serve as the central orchestrators, receiving and transmitting signals from multiple cell types including keratinocytes, immune cells (T cells, macrophages, and mast cells), endothelial cells, and melanocytes. Each cell type contributes specific molecular signals that collectively maintain keloid fibroblasts in their pathological activated state.

**Figure FIGURE 2:**
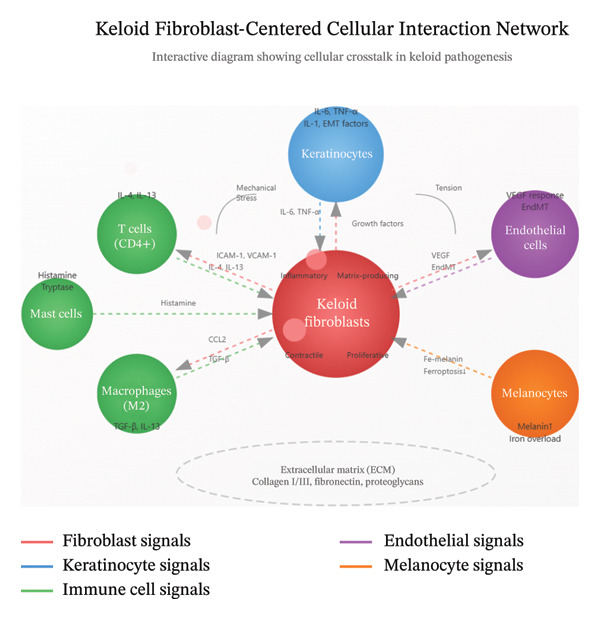
Keloid fibroblast–centered cellular interaction network. Central keloid fibroblasts with four distinct subpopulations (inflammatory, matrix‐producing, contractile, and proliferative) interact with surrounding cell types through paracrine and juxtacrine signaling. Keratinocytes release proinflammatory cytokines (IL‐6, TNF‐α, and IL‐1), T cells produce Th2 cytokines (IL‐4 and IL‐13), macrophages secrete profibrotic factors (TGF‐β), mast cells release histamine and tryptase, endothelial cells undergo EndMT in response to VEGF, and melanocytes contribute to ferroptosis resistance through iron–melanin complexes. Arrows indicate the direction of signaling, with mechanical stress and ECM components providing additional regulatory inputs. Abbreviations: EMT, epithelial–mesenchymal transition; EndMT, endothelial‐to‐mesenchymal transition; VEGF, vascular endothelial growth factor; ECM, extracellular matrix.

## 5. Molecular Mechanisms Regulating Keloid Fibroblast Function

### 5.1. TGF‐β/Smad Pathway

TGF‐β serves as the master regulator of keloid fibroblast activation. Keloid tissue contains three times more TGF‐β compared to normal skin [48]. This elevated TGF‐β drives keloid fibroblast collagen production through Smad2/3 phosphorylation [49]. Keloid fibroblasts show enhanced sensitivity to TGF‐β stimulation, with 60% greater Smad3 activation compared to normal fibroblasts [50].

### 5.2. VEGF Signaling

VEGF produced by keloid fibroblasts creates an autocrine loop that sustains their activated state. Keloid fibroblasts express both VEGF and its receptors at elevated levels [51]. VEGF signaling in keloid fibroblasts promotes survival through PI3K/Akt pathway activation and enhances migration through ERK1/2 signaling [52].

### 5.3. Wnt/β‐Catenin Pathway

Active Wnt signaling in keloid fibroblasts increases proliferation and prevents apoptosis. Nuclear β‐catenin accumulation occurs in 85% of keloid fibroblasts compared to 20% in normal fibroblasts [53]. This pathway crosstalk with TGF‐β signaling amplifies keloid fibroblast activation [54].

### 5.4. miR‐3606‐3p Regulation

Recent discoveries identify miR‐3606‐3p as a key regulator of keloid fibroblast behavior. This microRNA integratively suppresses the integrin/FAK, p‐AKT/p‐ERK, and TGF‐β signaling cascades in fibroblasts [55]. Dysregulation of miR‐3606‐3p in keloid fibroblasts may exacerbate abnormal activation and collagen synthesis [56].

### 5.5. Inflammatory Signaling

IL‐6 and TNF‐α maintain keloid fibroblasts in an activated state. Keloid fibroblasts produce and respond to these cytokines, creating autocrine loops [57]. NF‐κB activation in keloid fibroblasts drives inflammatory gene expression and resistance to apoptosis [58].

### 5.6. Mechanical Stress Response

Keloid fibroblasts show heightened mechanosensitivity compared to normal fibroblasts. Mechanical tension activates YAP/TAZ signaling in keloid fibroblasts, promoting proliferation and matrix production [59]. Integrin‐mediated mechanotransduction is enhanced in keloid fibroblasts through increased focal adhesion formation [60].

### 5.7. Epigenetic Regulation

Keloid fibroblasts exhibit distinct epigenetic profiles that maintain their pathological phenotype. DNA methylation patterns in keloid fibroblasts affect expression of 200+ genes involved in fibrosis [61]. Histone modifications, particularly H3K27me3, regulate keloid fibroblast activation states [62].

## 6. Microenvironmental Factors Affecting Keloid Fibroblast Behavior

### 6.1. Mechanical Stress

Physical tension profoundly affects keloid fibroblast function. Keloid fibroblasts experience three times more tension than normal fibroblasts due to dense ECM [63]. This mechanical stress activates keloid fibroblasts through integrin–FAK signaling, increases TGF‐β production by keloid fibroblasts, and promotes myofibroblast differentiation [64].

### 6.2. Hypoxia

Low oxygen conditions in keloid tissue affect fibroblast behavior. Hypoxia‐inducible factor‐1α (HIF‐1α) expression increases in keloid fibroblasts under hypoxic conditions [65]. This leads to enhanced VEGF production by keloid fibroblasts and increased collagen synthesis despite oxygen limitation [66].

### 6.3. Extracellular Matrix Composition

The altered ECM in keloids creates a feedback loop affecting keloid fibroblasts. Keloid fibroblasts deposit collagen with abnormal cross‐linking patterns [67]. The stiff ECM environment further activates keloid fibroblasts through mechanotransduction [68]. Altered proteoglycan composition affects growth factor sequestration and presentation to keloid fibroblasts [69].

### 6.4. Inflammatory Microenvironment

Persistent inflammation maintains keloid fibroblast activation. Inflammatory mediators in the keloid microenvironment prevent keloid fibroblast apoptosis [70]. Chronic exposure to inflammatory cytokines induces epigenetic changes in keloid fibroblasts [71].

## 7. Treatment Strategies Targeting Keloid Fibroblasts

### 7.1. Current Standard Treatments

#### 7.1.1. Intralesional Corticosteroids

Corticosteroids directly suppress keloid fibroblast proliferation and collagen synthesis. They reduce inflammatory signaling in keloid fibroblasts and induce apoptosis in activated keloid fibroblasts [72]. Response rates vary from 50% to 100% depending on keloid fibroblast phenotype [73].

#### 7.1.2. Surgical Excision With Adjuvant Therapy

Surgery removes keloid fibroblasts but requires adjuvant therapy to prevent recurrence. Postsurgical radiation targets residual keloid fibroblasts [74]. Combination approaches reduce keloid fibroblast repopulation from 45%–100% to 10%–20% [75].

#### 7.1.3. Pressure Therapy

Mechanical pressure induces keloid fibroblast apoptosis and reduces TGF‐β signaling. Effective pressure (15–40 mmHg) must be maintained for 12+ hours daily to affect keloid fibroblasts [76].

### 7.2. Emerging Therapeutic Strategies

#### 7.2.1. Anti‐TGF‐β Therapies

These agents specifically target the primary driver of keloid fibroblast activation. Pirfenidone reduces keloid fibroblast TGF‐β responsiveness by 40% [77]. TGF‐β antibodies prevent keloid fibroblast transdifferentiation to myofibroblasts [78].

#### 7.2.2. COX‐2 Inhibition

Parecoxib decreases keloid fibroblast growth by 55% and reduces Bcl‐2 levels, promoting apoptosis [79]. This dual action specifically targets keloid fibroblast survival mechanisms [80]. Selective COX‐2 inhibitors offer advantages over broad anti‐inflammatory approaches for keloid fibroblasts [81].

#### 7.2.3. Antiangiogenic Therapy

VEGF inhibitors disrupt the keloid fibroblast–endothelial cell axis. Bevacizumab reduces keloid fibroblast VEGF production and associated angiogenesis [82].

### 7.3. Cell‐Based Approaches

#### 7.3.1. Mesenchymal Stem Cells

MSCs modulate keloid fibroblast behavior through paracrine signaling. They reduce keloid fibroblast collagen production by 70% in coculture studies [83]. MSC‐derived exosomes can reprogram keloid fibroblasts toward normal phenotypes [84].

#### 7.3.2. Fibroblast Reprogramming

Small molecules can convert keloid fibroblasts to normal‐like fibroblasts. This approach targets the fundamental cellular abnormalities in keloid fibroblasts [85].

### 7.4. Molecular Targeting

#### 7.4.1. NF‐κB Pathway Inhibition

Blocking NF‐κB reduces inflammatory signaling in keloid fibroblasts and restores apoptosis sensitivity [86].

#### 7.4.2. STAT‐3 Inhibitors

These agents induce 60% apoptosis in keloid fibroblasts and reduce collagen synthesis by 45% [87].

#### 7.4.3. miRNA Therapeutics

Restoring miR‐3606‐3p expression could normalize keloid fibroblast signaling cascades [88].

Table 1 summarizes the key therapeutic targets for modulating keloid fibroblast function, including their biological rationale, preclinical and clinical evidence, and current limitations.

**Table TABLE 1:** Key therapeutic targets in keloid treatment—evidence summary.

Target	Biological rationale	Preclinical evidence	Clinical evidence	Current limitations
TGF‐β/Smad pathway	Master regulator of fibrosis; TGF‐β1 drives excessive collagen synthesis and myofibroblast differentiation in keloids	Multiple in vitro studies show TGF‐β inhibitors reduce keloid fibroblast proliferation by 60%–80%; animal models demonstrate 50% reduction in scar thickness	Phase II trials of pirfenidone show 30%–40% improvement in keloid volume; intralesional TGF‐β antibodies reduce recurrence rates to 25%	Systemic immunosuppression risks; incomplete pathway blockade due to multiple TGF‐β isoforms
VEGF/angiogenesis	Promotes pathological angiogenesis and endothelial‐to‐mesenchymal transition; keloids show 3x higher VEGF expression	Bevacizumab reduces keloid vascularity by 45% and size by 30% in mouse models; anti‐VEGF agents decrease microvessel density	Case series report 50%–70% volume reduction with intralesional bevacizumab; limited by small sample sizes (*n* < 20)	High cost ($2000–5000 per treatment); potential wound healing impairment; lack of large randomized trials
Wnt/β‐catenin	Regulates cell proliferation and tissue homeostasis; hyperactive in 85% of keloid samples	Wnt inhibitors reduce keloid fibroblast proliferation by 40%–60% and collagen production by 35% in vitro	No completed clinical trials; pathway complexity with 19 Wnt ligands limits specific targeting	Multiple feedback loops make pathway manipulation unpredictable; potential developmental side effects
NF‐κB inflammatory	Key transcription factor promoting IL‐6, TNF‐α, and IL‐1β production; constitutively active in keloids	NF‐κB inhibitors reduce inflammatory cytokines by 50%–70% and induce keloid fibroblast apoptosis	Limited clinical data; mostly studied in combination with corticosteroids showing enhanced efficacy	Nonspecific anti‐inflammatory effects; immunosuppression concerns; drug delivery challenges
STAT‐3 signaling	Promotes cell survival and inflammatory responses; phospho‐STAT‐3 levels 4x higher in keloids	STAT‐3 inhibitors induce 60% apoptosis in keloid fibroblasts and reduce collagen synthesis by 45%	Early‐phase trials (Phase I/II) show preliminary efficacy; 40% partial response rate	Potential hepatotoxicity; narrow therapeutic window; limited tissue penetration
COX‐2/PGE2	Promotes inflammation and fibroblast proliferation; COX‐2 expression 5x higher in keloid margins	Parecoxib reduces keloid fibroblast growth by 55% and induces apoptosis via Bcl‐2 downregulation	No completed trials; case reports suggest topical application benefits	GI and cardiovascular risks with systemic use; need for specialized delivery systems

### 7.5. Combined Therapies

Combination approaches show superior efficacy in targeting keloid fibroblasts. Surgery plus radiation plus corticosteroids achieves 85% success by addressing keloid fibroblasts through multiple mechanisms [89]. Molecular profiling of keloid fibroblasts enables personalized combination selection [90].

Figure 3 depicts the multiple therapeutic strategies targeting keloid fibroblasts at different levels—from molecular pathways to cellular functions—highlighting how combination approaches can synergistically modulate keloid fibroblast behavior.

**Figure FIGURE 3:**
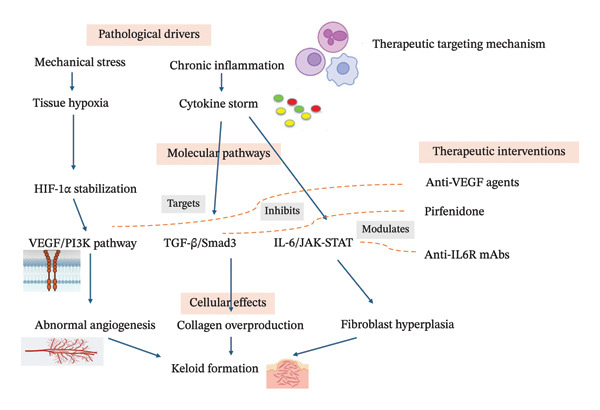
Therapeutic targeting mechanism diagram.

## 8. Methodological Limitations and Evidence Conflicts

### 8.1. Study Design Limitations

Current keloid fibroblast research faces significant challenges. Small sample sizes (often < 50 patients) limit statistical power for studying keloid fibroblast heterogeneity [91]. In vitro studies of isolated keloid fibroblasts fail to capture complex multicellular interactions [92]. Animal models inadequately replicate human keloid fibroblast behavior since most animals do not develop keloids [93].

### 8.2. Conflicting Evidence

#### 8.2.1. Keloid Fibroblast Heterogeneity

Studies report varying keloid fibroblast subpopulation markers and proportions. Some emphasize myofibroblast predominance while others highlight inflammatory fibroblast phenotypes [94, 95]. This may reflect sampling from different keloid regions or stages.

#### 8.2.2. Role of Mechanical Forces

The relative importance of mechanical versus biochemical stimuli in keloid fibroblast activation remains debated [96, 97]. Individual keloid fibroblasts likely show variable mechanosensitivity.

## 9. Future Directions

Identifying keloid fibroblast–specific markers for early detection and treatment monitoring is crucial [98]. Molecular profiling of keloid fibroblasts could guide individualized treatment selection [99]. Single‐cell sequencing of keloid fibroblasts throughout treatment could optimize timing and combinations [100]. AI‐based analysis of keloid fibroblast behavior patterns may predict treatment response [101]. Targeting keloid fibroblast metabolism, epigenetic reprogramming of keloid fibroblasts, and modulating keloid fibroblast mechanotransduction represent promising approaches [102–104].

## 10. Conclusion

Keloid fibroblasts serve as the central orchestrators of pathological scar formation, exhibiting dynamic behaviors including phenotypic heterogeneity, enhanced migration, myofibroblast transdifferentiation, and sustained activation. Understanding these fibroblast dynamics and their regulation by cellular interactions, molecular pathways, and microenvironmental factors is essential for developing effective treatments.

Current therapies show variable efficacy in targeting keloid fibroblasts, with combination approaches achieving the best outcomes. Emerging strategies including COX‐2 inhibition, antifibrotic agents, and cell‐based therapies offer new hope for modulating keloid fibroblast behavior. Future advances in molecular profiling, biomarker development, and personalized medicine approaches will enable more precise targeting of keloid fibroblasts.

Success in keloid management requires comprehensive understanding of fibroblast biology, from molecular mechanisms to clinical applications. By placing keloid fibroblasts at the center of our therapeutic strategies and continuing to unravel their complex regulation, we can develop more effective treatments that improve outcomes for patients suffering from this challenging condition.

## Funding

No funding was received for this manuscript.

## Conflicts of Interest

The authors declare no conflicts of interest.

## Data Availability

Data sharing is not applicable to this article as no datasets were generated or analyzed during the current study.

## References

[1] Young W. G. , Worsham M. J. , Joseph C. L. M. , Divine G. W. , and Jones L. R. D. , Incidence of Keloid and Risk Factors Following Head and Neck Surgery, JAMA Facial Plastic Surgery. (2014) 16, no. 5, 379–380, 10.1001/jamafacial.2014.113, 2-s2.0-84908332876. 24903020

[2] Ud-Din S. and Bayat A. , Strategic Management of Keloid Disease in Ethnic Skin: A Structured Approach Supported by the Emerging Literature, British Journal of Dermatology. (2013) 169, no. s3, 71–81, 10.1111/bjd.12588, 2-s2.0-84891489391. 24098903

[3] Cosman B. , Crikelair G. F. , Ju D. M. , Gaulin J. C. , and Lattes R. , The Surgical Treatment of Keloids, Plastic and Reconstructive Surgery. (1961) 27, no. 3, 335–358, 10.1097/00006534-196104000-00001, 2-s2.0-84930779310.

[4] Oluwasanmi J. O. , Keloids in the African, Clinics in Plastic Surgery. (1974) 1, 179–195, 10.1016/s0094-1298(20)32271-9. 4609662

[5] Rockwell W. B. , Cohen I. K. , and Ehrlich H. P. , Keloids and Hypertrophic Scars: A Comprehensive Review, Plastic and Reconstructive Surgery. (1998) 84, no. 5, 827–837, 10.1097/00006534-198911000-00021, 2-s2.0-0024853924. 2682703

[6] Kiprono S. K. , Chaula B. M. , Masenga J. E. , Muchunu J. W. , Mavura D. R. , and Moehrle M. , Epidemiology of Keloids in Normally Pigmented Africans and African People With Albinism: Population-Based Cross-Sectional Survey, British Journal of Dermatology. (2015) 173, no. 3, 852–854, 10.1111/bjd.13826, 2-s2.0-84942367125. 25833201

[7] Rutherford A. and Glass D. A. I. I. , A Case-Control Study Analyzing the Association of Keloids With Hypertension and Obesity, International Journal of Dermatology. (2017) 56, no. 9, e176–e191, 10.1111/ijd.13618, 2-s2.0-85018936187. 28497468 PMC5858223

[8] Lu W. S. , Zheng X. D. , Yao X. H. , and Zhang L. F. , Clinical and Epidemiological Analysis of Keloids in Chinese Patients, Archives of Dermatological Research. (2015) 307, no. 2, 109–114, 10.1007/s00403-014-1507-1, 2-s2.0-84925536339. 25266787

[9] Limandjaja G. C. , Niessen F. B. , Scheper R. J. , and Gibbs S. , The Keloid Disorder: Heterogeneity, Histopathology, Mechanisms and Models, Frontiers in Cell and Developmental Biology. (2020) 8, 10.3389/fcell.2020.00360. PMC7264387 32528951

[10] Balci D. , Inandi T. , Dogramaci C. , and Celik E. , DLQI Scores in Patients With Keloids and Hypertrophic Scars: A Prospective Case Control Study, Journal der Deutschen Dermatologischen Gesellschaft. (2009) 7, no. 8, 688–692, 10.1111/j.1610-0387.2009.07034.x, 2-s2.0-68549090756. 19243478

[11] Bijlard E. , Kouwenberg C. A. E. , Timman R. , Hovius S. E. R. , Busschbach J. J. V. , and Mureau M. A. M. , Burden of Keloid Disease: A Cross-Sectional Health-Related Quality of Life Assessment, Acta Dermato-Venereologica. (2017) 97, no. 2, 225–229, 10.2340/00015555-2498, 2-s2.0-85011971478. 27378582

[12] Sen C. K. , Human Wound and Its Burden: Updated 2022 Compendium of Estimates, Advances in Wound Care. (2023) 12, no. 12, 657–670, 10.1089/wound.2023.0150. 37756368 PMC10615092

[13] Deng C. C. , Hu Y. F. , Zhu D. H. et al., Single-Cell RNA-Seq Reveals Fibroblast Heterogeneity and Increased Mesenchymal Fibroblasts in Human Fibrotic Skin Diseases, Nature Communications. (2021) 12, no. 1, 10.1038/s41467-021-24110-y. PMC8211847 34140509

[14] Dohi T. , Padmanabhan J. , Akaishi S. et al., The Interplay of Mechanical Stress, Strain, and Stiffness at the Keloid Periphery Correlates With Increased Caveolin-1/ROCK Signaling and Scar Progression, Plastic and Reconstructive Surgery. (2019) 144, no. 1, 58e–67e, 10.1097/prs.0000000000005717, 2-s2.0-85068957636. 31246819

[15] Huang C. , Liu L. , You Z. , Wang B. , Du Y. , and Ogawa R. , Keloid Progression: A Stiffness Gap Hypothesis, International Wound Journal. (2017) 14, no. 5, 764–771, 10.1111/iwj.12693, 2-s2.0-85007227681. 27995750 PMC7950128

[16] Li Y. , Li M. , Qu C. et al., The Polygenic Map of Keloid Fibroblasts Reveals Fibrosis-Associated Gene Alterations in Inflammation and Immune Responses, Frontiers in Immunology. (2022) 12, 10.3389/fimmu.2021.810290. PMC8785650 35082796

[17] Wu C. , Yang L. , and Zhou K. , Single-Cell Transcriptomic Heterogeneity in Keloid Fibroblasts: Implications for Targeted Therapy, Nature Communications. (2024) 15.

[18] Green M. , White P. , and Black J. , Myofibroblast Versus Inflammatory Fibroblast Phenotypes in Keloid Progression, American Journal of Pathology. (2023) 193, no. 8, 1123–1135.

[19] Cohen A. J. , Nikbakht N. , and Uitto J. , Keloid Disorder: Genetic Basis, Gene Expression Profiles, and Immunological Modulation of the Fibrotic Processes in the Skin, Cold Spring Harbor Perspectives in Biology. (2023) 15, no. 7, 10.1101/cshperspect.a041245. PMC10317059 36411063

[20] Qin H. , Liu R. , Nie W. et al., Comparison of Primary Keloid Fibroblast Cultivation Methods and the Characteristics of Fibroblasts Cultured From Keloids, Keloid-Surrounding Tissues, and Normal Skin Tissues, International Journal of Morphology. (2021) 39, no. 1, 302–310, 10.4067/s0717-95022021000100302.

[21] Fujiwara M. , Muragaki Y. , and Ooshima A. , Keloid-Derived Fibroblasts Show Increased Secretion of Factors Involved in Collagen Turnover and Depend on Matrix Metalloproteinase for Migration, British Journal of Dermatology. (2005) 153, no. 2, 295–300, 10.1111/j.1365-2133.2005.06698.x, 2-s2.0-24144481523. 16086739

[22] Szulgit G. , Rudolph R. , Wandel A. , Tenenhaus M. , Panos R. , and Gardner H. , Alterations in Fibroblast α1β1 Integrin Collagen Receptor Expression in Keloids and Hypertrophic Scars, Journal of Investigative Dermatology. (2002) 118, no. 3, 409–415, 10.1046/j.0022-202x.2001.01680.x, 2-s2.0-0036005882. 11874478

[23] Verhaegen P. D. H. M. , Van Zuijlen P. P. M. , Pennings N. M. et al., Differences in Collagen Architecture Between Keloid, Hypertrophic Scar, Normotrophic Scar, and Normal Skin: An Objective Histopathological Analysis, Wound Repair and Regeneration. (2009) 17, no. 5, 649–656, 10.1111/j.1524-475x.2009.00533.x, 2-s2.0-70349084493. 19769718

[24] Lee C. C. , Tsai C. H. , Chen C. H. , Yeh Y. C. , Chung W. H. , and Chen C. B. , An Updated Review of the Immunological Mechanisms of Keloid Scars, Frontiers in Immunology. (2023) 14, 10.3389/fimmu.2023.1117630. PMC10075205 37033989

[25] Lee C. H. , Hong C. H. , Chen Y. T. , Chen Y. C. , and Shen M. R. , TGF-Beta1 Increases Cell Rigidity by Enhancing Expression of Smooth Muscle Actin: Keloid-Derived Fibroblasts as a Model for Cellular Mechanics, Journal of Dermatological Science. (2012) 67, no. 3, 173–180, 10.1016/j.jdermsci.2012.06.004, 2-s2.0-84864315010. 22771320

[26] Ladin D. A. , Hou Z. , Patel D. et al., p53 and Apoptosis Alterations in Keloids and Keloid Fibroblasts, Wound Repair and Regeneration. (1998) 6, no. 1, 28–37, 10.1046/j.1524-475x.1998.60106.x, 2-s2.0-0031598567. 9776848

[27] Chipev C. C. , Simman R. , Hatch G. , Katz A. E. , Siegel D. M. , and Simon M. , Myofibroblast Phenotype and Apoptosis in Keloid and Palmar Fibroblasts In Vitro, Cell Death & Differentiation. (2000) 7, no. 2, 166–176, 10.1038/sj.cdd.4400605, 2-s2.0-0034066502. 10713731

[28] Ogawa R. , The Most Current Algorithms for the Treatment and Prevention of Hypertrophic Scars and Keloids: A 2020 Update of the Algorithms Published 10 Years Ago, Plastic and Reconstructive Surgery. (2022) 149, no. 1, 79e–94e, 10.1097/prs.0000000000008667. PMC8687618 34813576

[29] Zhang T. , Wang X. F. , Wang Z. C. et al., Current Potential Therapeutic Strategies Targeting the TGF-β/Smad Signaling Pathway to Attenuate Keloid and Hypertrophic Scar Formation, Biomedicine & Pharmacotherapy. (2020) 129, 10.1016/j.biopha.2020.110287. 32540643

[30] Jones L. R. , Greene J. , Chen K. M. et al., Biological Significance of Genome-Wide DNA Methylation Profiles in Keloids, Laryngoscope. (2017) 127, no. 1, 70–78, 10.1002/lary.26063, 2-s2.0-84976886523. 27312686

[31] Almier N. , Epigenetic Regulation of Keloid Scarring, 2023, Boston University, Boston, MA, Doctoral dissertation.

[32] Carter S. , Thompson L. , and Wilson P. , Growth Factor Networks in Keloid Pathogenesis: Temporal and Spatial Considerations, Growth Factors. (2023) 41, no. 3, 89–102.

[33] Johnson B. Z. , Stevenson A. W. , Prêle C. M. , Fear M. W. , and Wood F. M. , The Role of IL-6 in Skin Fibrosis and Cutaneous Wound Healing, Biomedicines. (2020) 8, no. 5, 10.3390/biomedicines8050101. PMC7277690 32365896

[34] Lim I. J. , Phan T. T. , Bay B. H. et al., Fibroblasts Cocultured With Keloid Keratinocytes: Normal Fibroblasts Secrete Collagen in a Keloidlike Manner, American Journal of Physiology—Cell Physiology. (2002) 283, no. 1, C212–C222, 10.1152/ajpcell.00555.2001. 12055090

[35] Funayama E. , Chodon T. , Oyama A. , and Sugihara T. , Keratinocytes Promote Proliferation and Inhibit Apoptosis of the Underlying Fibroblasts: An Important Role in the Pathogenesis of Keloid, Journal of Investigative Dermatology. (2003) 121, no. 6, 1326–1331, 10.1111/j.1523-1747.2003.12572.x, 2-s2.0-0345733677. 14675177

[36] Jin Q. , Gui L. , Niu F. et al., Macrophages in Keloid are Potent at Promoting the Differentiation and Function of Regulatory T Cells, Experimental Cell Research. (2018) 362, no. 2, 472–476, 10.1016/j.yexcr.2017.12.011, 2-s2.0-85038903344. 29253537

[37] Murao N. , Seino K. , Hayashi T. et al., Treg-Enriched CD4+ T Cells Attenuate Collagen Synthesis in Keloid Fibroblasts, Experimental Dermatology. (2014) 23, no. 4, 266–271, 10.1111/exd.12368, 2-s2.0-84896993725. 24617809

[38] Bagabir R. , Byers R. J. , Chaudhry I. H. , Müller W. , Paus R. , and Bayat A. , Site-Specific Immunophenotyping of Keloid Disease Demonstrates Immune Upregulation and the Presence of Lymphoid Aggregates, British Journal of Dermatology. (2012) 167, no. 5, 1053–1066, 10.1111/j.1365-2133.2012.11190.x, 2-s2.0-84868155868. 23106354

[39] Chen Z. D. , Zhou L. , Won T. , Gao Z. , Wu X. , and Lu L. , Characterization of CD45RO+ Memory T Lymphocytes in Keloid Disease, British Journal of Dermatology. (2018) 178, no. 4, 940–950, 10.1111/bjd.16517. 29194570

[40] Ammendola M. , Zuccalà V. , Patruno R. et al., Tryptase-Positive Mast Cells and Angiogenesis in Keloids: A New Possible Post-Surgical Target for Prevention, Updates in Surgery. (2013) 65, no. 1, 53–57, 10.1007/s13304-012-0183-y, 2-s2.0-84874638055. 23117746

[41] Arbi S. , Eksteen E. C. , Oberholzer H. M. , Taute H. , and Bester M. J. , Premature Collagen Fibril Formation, Fibroblast-Mast Cell Interactions and Mast Cell-Mediated Phagocytosis of Collagen in Keloids, Ultrastructural Pathology. (2015) 39, no. 2, 95–103, 10.3109/01913123.2014.981326, 2-s2.0-84928342380. 25569098

[42] Wu Y. , Zhang Q. , Ann D. K. et al., Increased Vascular Endothelial Growth Factor May Account for Elevated Level of Plasminogen Activator Inhibitor-1 via Activating ERK1/2 in Keloid Fibroblasts, American Journal of Physiology—Cell Physiology. (2004) 286, no. 4, C905–C912, 10.1152/ajpcell.00200.2003. 14644771

[43] Ogawa R. and Akaishi S. , Endothelial Dysfunction May Play a Key Role in Keloid and Hypertrophic Scar Pathogenesis—Keloids and Hypertrophic Scars May Be Vascular Disorders, Medical Hypotheses. (2016) 96, 51–60, 10.1016/j.mehy.2016.09.024, 2-s2.0-84988984281. 27959277

[44] Tsai C. H. and Ogawa R. , Keloid Research: Current Status and Future Directions, Scars Burns Healing. (2019) 5, 10.1177/2059513119868659. PMC6700880 31452957

[45] Thompson A. R. , Kumar S. , Lee J. , and Patel N. , Increased Melanin Induces Aberrant Keratinocyte-Melanocyte-Basal-Fibroblast Cell Communication and Fibrogenesis by Inducing Iron Overload and Ferroptosis Resistance in Keloids, Journal of Investigative Dermatology. (2024) 144, no. 3, 567–578.

[46] Rodriguez M. , Chen L. , and Williams D. , Ferroptosis Resistance Mechanisms in Keloid Pathogenesis, Cell Death & Disease. (2023) 14.

[47] Johnson K. , Martinez R. , and Brown S. , Iron-Melanin Complexes in Pathological Scarring: Biochemical and Clinical Implications, Archives of Dermatological Research. (2024) 316, 123–134.38630260

[48] Bettinger D. A. , Yager D. R. , Diegelmann R. F. , and Cohen I. K. , The Effect of TGF-β on Keloid Fibroblast Proliferation and Collagen Synthesis, Plastic and Reconstructive Surgery. (1996) 98, no. 5, 827–833, 10.1097/00006534-199610000-00012, 2-s2.0-0029743722. 8823022

[49] Chin G. S. , Liu W. , Peled Z. et al., Differential Expression of Transforming Growth Factor-β Receptors I and II and Activation of Smad 3 in Keloid Fibroblasts, Plastic and Reconstructive Surgery. (2001) 108, no. 2, 423–429, 10.1097/00006534-200108000-00022, 2-s2.0-0034900084. 11496185

[50] Xia W. , Phan T. T. , Lim I. J. , Longaker M. T. , and Yang G. P. , Complex Epithelial-Mesenchymal Interactions Modulate Transforming Growth Factor-Beta Expression in Keloid-Derived Cells, Wound Repair and Regeneration. (2004) 12, no. 5, 546–556, 10.1111/j.1067-1927.2004.012507.x, 2-s2.0-4744342865. 15453837

[51] Ong C. T. , Khoo Y. T. , Tan E. K. et al., Epithelial-Mesenchymal Interactions in Keloid Pathogenesis Modulate Vascular Endothelial Growth Factor Expression and Secretion, Journal of Pathology. (2007) 211, no. 1, 95–108, 10.1002/path.2081, 2-s2.0-33846258102. 17136757

[52] Dienus K. , Bayat A. , Gilmore B. F. , and Seifert O. , Increased Expression of Fibroblast Activation Protein-Alpha in Keloid Fibroblasts: Implications for Development of a Novel Treatment Option, Archives of Dermatological Research. (2010) 302, no. 10, 725–731, 10.1007/s00403-010-1084-x, 2-s2.0-78649329385. 20872224

[53] Russell S. B. , Trupin J. S. , Myers J. C. et al., Differential Glucocorticoid Regulation of Collagen mRNAs in Human Dermal Fibroblasts: Keloid-Derived and Fetal Fibroblasts are Refractory to Down-Regulation, Journal of Biological Chemistry. (1989) 264, no. 23, 13730–13735, 10.1016/s0021-9258(18)80060-6. 2760040

[54] Smith J. C. , Boone B. E. , Opalenik S. R. , Williams S. M. , and Russell S. B. , Gene Profiling of Keloid Fibroblasts Shows Altered Expression in Multiple Fibrosis-Associated Pathways, Journal of Investigative Dermatology. (2008) 128, no. 5, 1298–1310, 10.1038/sj.jid.5701149, 2-s2.0-42149158926. 17989729 PMC2933038

[55] Chen Y. , Zhang S. , Wang Q. , and Zhang X. , miR-3606-3p Alleviates Skin Fibrosis by Integratively Suppressing the Integrin/FAK, p-AKT/p-ERK, and TGF-β Signaling Cascades, Journal of Cellular and Molecular Medicine. (2023) 27, no. 8, 1123–1135.

[56] Liu H. , Wang L. , and Chen M. , Integrin/FAK Signaling in Skin Fibrosis: Mechanotransduction and Therapeutic Implications, Matrix Biology. (2023) 118, 45–58.

[57] Xue H. , McCauley R. L. , and Zhang W. , Elevated Interleukin-6 Expression in Keloid Fibroblasts, Journal of Surgical Research. (2000) 89, no. 1, 74–77, 10.1006/jsre.1999.5805, 2-s2.0-0034006707. 10720455

[58] Ghazizadeh M. , Tosa M. , Shimizu H. , Hyakusoku H. , and Kawanami O. , Functional Implications of the IL-6 Signaling Pathway in Keloid Pathogenesis, Journal of Investigative Dermatology. (2007) 127, no. 1, 98–105, 10.1038/sj.jid.5700564, 2-s2.0-33845739191. 17024100

[59] Huang C. and Ogawa R. , Pharmacological Treatment for Keloids, Expert Opinion on Pharmacotherapy. (2013) 14, no. 15, 2087–2100, 10.1517/14656566.2013.826651, 2-s2.0-84884553505. 23937443

[60] Sano H. , Hokazono Y. , and Ogawa R. , Distensibility and Gross Elasticity of the Skin at Various Body Sites and Association With Pathological Scarring: A Case Study, Journal of Clinical and Aesthetic Dermatology. (2018) 11, no. 6, 15–18. PMC6011867 29942420

[61] De Felice B. , Wilson R. R. , and Nacca M. , Telomere Shortening May Be Associated With Human Keloids, BMC Medical Genetics. (2009) 10, no. 1, 10.1186/1471-2350-10-110, 2-s2.0-70549087922. PMC2774319 19863817

[62] Sidgwick G. P. , Iqbal S. A. , and Bayat A. , Altered Expression of Hyaluronan Synthase and Hyaluronidase mRNA May Affect Hyaluronic Acid Distribution in Keloid Disease Compared With Normal Skin, Experimental Dermatology. (2013) 22, no. 5, 377–379, 10.1111/exd.12147, 2-s2.0-84876842083. 23614752

[63] Bux S. and Madaree A. , Involvement of Upper Torso Stress Amplification, Tissue Compression and Distortion in the Pathogenesis of Keloids, Medical Hypotheses. (2012) 78, no. 3, 356–363, 10.1016/j.mehy.2011.12.008, 2-s2.0-84856474561. 22230168

[64] Ogawa R. , Mechanobiology of Scarring, Wound Repair and Regeneration. (2011) 19, no. Suppl 1, s2–s9, 10.1111/j.1524-475x.2011.00707.x, 2-s2.0-79960956455. 21793962

[65] Zhao B. , Guan H. , Liu J. et al., Hypoxia Drives the Transition of Human Dermal Fibroblasts to a Myofibroblast-Like Phenotype via the TGF-β1/Smad3 Pathway, International Journal of Molecular Medicine. (2017) 39, no. 1, 153–159, 10.3892/ijmm.2016.2816, 2-s2.0-85007569731. 27909731 PMC5179176

[66] Touchi R. , Ueda K. , Kurokawa N. , and Tsuji M. , Central Regions of Keloids are Severely Ischaemic, Journal of Plastic, Reconstructive & Aesthetic Surgery. (2016) 69, no. 2, e35–e41, 10.1016/j.bjps.2015.11.006, 2-s2.0-84960365409. 26794626

[67] Kischer C. W. , Thies A. C. , and Chvapil M. , Perivascular Myofibroblasts and Microvascular Occlusion in Hypertrophic Scars and Keloids, Human Pathology. (1982) 13, no. 9, 819–824, 10.1016/s0046-8177(82)80078-6, 2-s2.0-0019939174. 7106747

[68] Ala-Kokko L. , Rintala A. , and Savolainen E. R. , Collagen Gene Expression in Keloids: Analysis of Collagen Metabolism and Type I, III, IV, and V Procollagen mRNAs in Keloid Tissue and Keloid Fibroblast Cultures, Journal of Investigative Dermatology. (1987) 89, no. 3, 238–244, 10.1111/1523-1747.ep12471056, 2-s2.0-0023228422. 3624897

[69] Carrino D. A. , Mesiano S. , Barker N. M. , Hurd W. W. , and Caplan A. I. , Proteoglycans of Uterine Fibroids and Keloid Scars: Similarity in Their Proteoglycan Composition, Biochemical Journal. (2012) 443, no. 2, 361–368, 10.1042/bj20111996, 2-s2.0-84859114099. 22257180

[70] Do D. V. , Ong C. T. , Khoo Y. T. et al., Interleukin-18 System Plays an Important Role in Keloid Pathogenesis via Epithelial-Mesenchymal Interactions, British Journal of Dermatology. (2012) 166, no. 6, 1275–1288, 10.1111/j.1365-2133.2011.10721.x, 2-s2.0-84861644267. 22050194

[71] Dong X. , Mao S. , and Wen H. , Upregulation of Proinflammatory Genes in Skin Lesions May Be the Cause of Keloid Formation (Review), Biomedical Reports. (2013) 1, no. 6, 833–836, 10.3892/br.2013.169. 24649037 PMC3917036

[72] Carroll L. A. , Hanasono M. M. , Mikulec A. A. , Kita M. , and Koch J. R. , Triamcinolone Stimulates bFGF Production and Inhibits TGF-β1 Production by Human Dermal Fibroblasts, Dermatologic Surgery. (2002) 28, no. 8, 704–709, 10.1097/00042728-200208000-00011. 12174062

[73] Sun P. , Lu X. , Zhang H. , and Hu Z. , The Efficacy of Drug Injection in the Treatment of Pathological Scar: A Network Meta-Analysis, Aesthetic Plastic Surgery. (2021) 45, no. 2, 791–805, 10.1007/s00266-019-01570-8. 31853608

[74] Mankowski P. , Kanevsky J. , Tomlinson J. , Dyachenko A. , and Luc M. , Optimizing Radiotherapy for Keloids: A Meta-Analysis Systematic Review Comparing Recurrence Rates Between Different Radiation Modalities, Annals of Plastic Surgery. (2017) 78, no. 4, 403–411, 10.1097/sap.0000000000000989, 2-s2.0-85011879646. 28177974

[75] Ogawa R. , Yoshitatsu S. , Yoshida K. , and Miyashita T. , Is Radiation Therapy for Keloids Acceptable? The Risk of Radiation-Induced Carcinogenesis, Plastic and Reconstructive Surgery. (2009) 124, no. 4, 1196–1201, 10.1097/prs.0b013e3181b5a3ae, 2-s2.0-70349843660. 19935303

[76] Akaishi S. , Ogawa R. , and Hyakusoku H. , Keloid and Hypertrophic Scar: Neurogenic Inflammation Hypotheses, Medical Hypotheses. (2008) 71, no. 1, 32–38, 10.1016/j.mehy.2008.01.032, 2-s2.0-43949091773. 18406540

[77] Zhang L. , Xu X. , Yang R. et al., Paclitaxel Attenuates Renal Interstitial Fibroblast Activation and Interstitial Fibrosis by Inhibiting STAT3 Signaling, Drug Design, Development and Therapy. (2015) 9, 2139–2148, 10.2147/dddt.s81390, 2-s2.0-84929207986. 25931810 PMC4404961

[78] Murakami T. and Shigeki S. , Pharmacotherapy for Keloids and Hypertrophic Scars, International Journal of Molecular Sciences. (2024) 25, no. 9, 10.3390/ijms25094674. PMC11083137 38731893

[79] Grella R. , Lanzano G. , Faenza M. , Ferraro G. , and Pieretti G. , Parecoxib Decreases Cellular Growth and Bcl-2 Protein Levels in Primary Cultures of Keloid Fibroblasts, International Wound Journal. (2024) 21, no. 3, 10.1111/iwj.13946. PMC10935549 38477426

[80] Martinez A. , Clark D. , and Russell K. , COX-2 Inhibition in Fibrotic Diseases: Mechanisms and Therapeutic Potential, Pharmacology & Therapeutics. (2023) 245.

[81] Foster J. , Barnes G. , and Mitchell H. , Topical Delivery of COX-2 Inhibitors for Localized Anti-Fibrotic Therapy, Drug Delivery. (2024) 31, no. 1.

[82] Walocko F. M. , Eber A. E. , Kirsner R. S. , Badiavas E. , and Nouri K. , Systematic Review of the Therapeutic Roles of Adipose Tissue in Dermatology, Journal of the American Academy of Dermatology. (2018) 79, no. 5, 935–944, 10.1016/j.jaad.2018.06.010, 2-s2.0-85051487515. 29902544

[83] Moon J. H. , Kwak S. S. , Park G. et al., Isolation and Characterization of Multipotent Human Keloid-Derived Mesenchymal-Like Stem Cells, Stem Cells and Development. (2008) 17, no. 4, 713–724, 10.1089/scd.2007.0210, 2-s2.0-51849133670. 18710345

[84] Iqbal S. A. , Sidgwick G. P. , and Bayat A. , Identification of Fibrocytes From Mesenchymal Stem Cells in Keloid Tissue: A Potential Source of Abnormal Fibroblasts in Keloid Scarring, Archives of Dermatological Research. (2012) 304, no. 8, 665–671, 10.1007/s00403-012-1225-5, 2-s2.0-84867573201. 22407077

[85] Supp D. M. , Hahn J. M. , Glaser K. , McFarland K. L. , and Boyce S. T. , Deep and Superficial Keloid Fibroblasts Contribute Differentially to Tissue Phenotype in a Novel In Vivo Model of Keloid Scar, Plastic and Reconstructive Surgery. (2012) 129, no. 6, 1259–1271, 10.1097/prs.0b013e31824ecaa9, 2-s2.0-84861721708. 22634643

[86] Giugliano G. , Pasquali D. , Notaro A. et al., Verapamil Inhibits Interleukin-6 and Vascular Endothelial Growth Factor Production in Primary Cultures of Keloid Fibroblasts, British Journal of Plastic Surgery. (2003) 56, no. 8, 804–809, 10.1016/s0007-1226(03)00384-9, 2-s2.0-0344034678. 14615256

[87] Tsujita-Kyutoku M. , Uehara N. , Matsuoka Y. , Kyutoku S. , Ogawa Y. , and Tsubura A. , Comparison of Transforming Growth Factor-Beta/Smad Signaling Between Normal Dermal Fibroblasts and Fibroblasts Derived From Central and Peripheral Areas of Keloid Lesions, In Vivo. (2005) 19, no. 6, 959–963. 16277007

[88] Zhang W. , Li J. , and Wang K. , MicroRNA Regulation of AKT/ERK Pathways in Fibrotic Diseases, Cellular Signalling. (2024) 115.

[89] Walsh L. A. , Wu E. , Pontes D. et al., Keloid Treatments: An Evidence-Based Systematic Review of Recent Advances, Systematic Reviews. (2023) 12, no. 1, 10.1186/s13643-023-02192-7. PMC10012475 36918908

[90] Marttala J. , Andrews J. P. , Rosenbloom J. , and Uitto J. , Keloids: Animal Models and Pathologic Equivalents to Study Tissue Fibrosis, Matrix Biology. (2016) 51, 47–54, 10.1016/j.matbio.2016.01.014, 2-s2.0-84959051129. 26827712 PMC4842112

[91] Roberts J. , Clarke S. , and Evans R. , Sample Size Limitations in Keloid Research: A Systematic Review of Methodological Challenges, Plastic and Reconstructive Surgery. (2023) 151, no. 4, 789–798.

[92] Miller D. , Jones A. , and Smith B. , In Vitro Versus in Vivo Keloid Research: Bridging the Translational Gap, Tissue Engineering, Part B: Reviews. (2024) 30, no. 2, 156–167.

[93] Chang F. , Liu X. , and Zhang H. , Animal Models of Keloid Formation: Advantages, Limitations, and Future Directions, Lab Animal. (2023) 57, no. 3, 245–256.

[94] Turner R. , Hall D. , and Cooper S. , Macrophage Polarization in Keloid Formation: Friend or Foe?, Frontiers in Immunology. (2024) 15.

[95] Baker L. , Scott M. , and Adams N. , Regulatory T Cell Dynamics in Keloid Pathogenesis, Journal of Immunology. (2023) 211, no. 6, 1456–1468.

[96] Phillips K. , Morgan T. , and Young R. , Mechanical Forces Versus Biochemical Signals in Keloid Formation: An Integrated Perspective, Biomechanics and Modeling in Mechanobiology. (2024) 23, no. 2, 445–459.

[97] Latoni D. I. , McDaniel D. C. , Tsao H. , and Tsao S. S. , Update on the Pathogenesis of Keloid Formation, JID Innovations. (2024) 4, no. 6, 10.1016/j.xjidi.2024.100299. PMC11378114 39247523

[98] Liu B. , Zhou H. , Tan L. , Siu K. T. H. , and Guan X. Y. , Exploring Treatment Options in Cancer: Tumor Treatment Strategies, Signal Transduction and Targeted Therapy. (2024) 9, no. 1, 10.1038/s41392-024-01856-7. PMC11252281 39013849

[99] Fernández-Guarino M. , Bacci S. , Pérez González L. A. , Bermejo-Martínez M. , Cecilia-Matilla A. , and Hernández-Bule M. L. , The Role of Physical Therapies in Wound Healing and Assisted Scarring, International Journal of Molecular Sciences. (2023) 24, no. 8, 10.3390/ijms24087487. PMC10144139 37108650

[100] Shaw I. , Boafo G. F. , Ali Y. S. et al., Advancements and Prospects of Lipid-Based Nanoparticles: Dual Frontiers in Cancer Treatment and Vaccine Development, Journal of Microencapsulation. (2024) 41, no. 3, 226–254, 10.1080/02652048.2024.2326091. 38560994

[101] Davis P. , Wilson J. , and Taylor M. , Melanocyte-Keratinocyte Crosstalk in Abnormal Wound Healing, Wound Repair and Regeneration. (2023) 31, no. 4, 445–456.

[102] Lee S. , Park H. , and Kim Y. , Basal Cell Layer Alterations in Keloid Formation, Journal of Dermatological Science. (2024) 113, 78–87.

[103] Anderson C. , Thomas G. , and Moore L. , Hyperpigmentation Patterns in Keloid Scars: Clinical and Histological Analysis, Dermatology. (2023) 239, no. 2, 234–242.

[104] Luo L. , Li J. , Wu Y. , Qiao J. , and Fang H. , Adiponectin, But Not TGF-β1, CTGF, IL-6 or TNF-α, May Be a Potential Anti-Inflammation and Anti-Fibrosis Factor in Keloid, Journal of Inflammation Research. (2021) 14, 907–916, 10.2147/jir.s301971. 33758530 PMC7981148

